# The role of prostate-specific membrane antigen PET/computed tomography in the management of prostate cancer patients: could we ask for more?

**DOI:** 10.1097/MOU.0000000000000982

**Published:** 2022-05-11

**Authors:** Riccardo Mei, Andrea Farolfi, Joshua James Morigi, Stefano Fanti

**Affiliations:** a Nuclear Medicine, IRCCS, Azienda Ospedaliero-Universitaria di Bologna; b DIMES, University of Bologna, Bologna, Italy; c PET/CT Unit, Department of Medical Imaging, Royal Darwin Hospital, Darwin, Australia

**Keywords:** androgen deprivation therapy, castrate-resistant prostate cancer, prostate cancer, prostate-specific membrane antigen PET/CT/CT, radioligand therapy, staging

## Abstract

**Purpose of review:**

Thanks to the development of novel PSMA-based peptides, molecular imaging, such as PET/CT paired with theranostic-based approaches have recently been proposed for treatment of prostate cancer. Patient selection, however, remains challenging because of the absence of strong prospective data to interpret and translate imaging scans into effective and well tolerated treatment regimens.

**Recent findings:**

In this review, we discuss the latest findings in PSMA imaging in prostate cancer patients. Particularly, we go into detail into the impact of PSMA imaging on the treatment management in primary staging, biochemical recurrence and in advanced prostate cancer.

**Summary:**

For primary prostate cancer staging, PSMA PET/CT seems crucial for primary therapy assessment, being able in some cases to detect lesions outside the surgical template, thus permitting a change in management. Moreover, N+ condition at PSMA has been correlated with a worse biochemical recurrence-free and therapy-free survival. The early detection of PSMA-positive findings in recurrent prostate cancer is associated with a better time to relapse survival. Similarly, for advanced prostate cancer patients, accurate restaging with PSMA imaging is gaining importance for early prediction of response to systemic therapies and to assure the best outcome possible. With regards to theranostics, appropriate selection of patients eligible for ^177^Lu-PSMA requires PSMA imaging, whereas the role of added FDG-PET for discriminating those with PSMA/FDG discordance needs to be further evaluated.

## INTRODUCTION

Prostate cancer is one of the most frequent malignancies in men. Although frequently indolent, nearly 25% of prostate cancer patients present with high-risk disease, potentially leading to poorer outcomes [1]. Accurate staging of the extension of the tumor is, therefore, pivotal for immediate or future treatment decisions. 

**Box 1:**
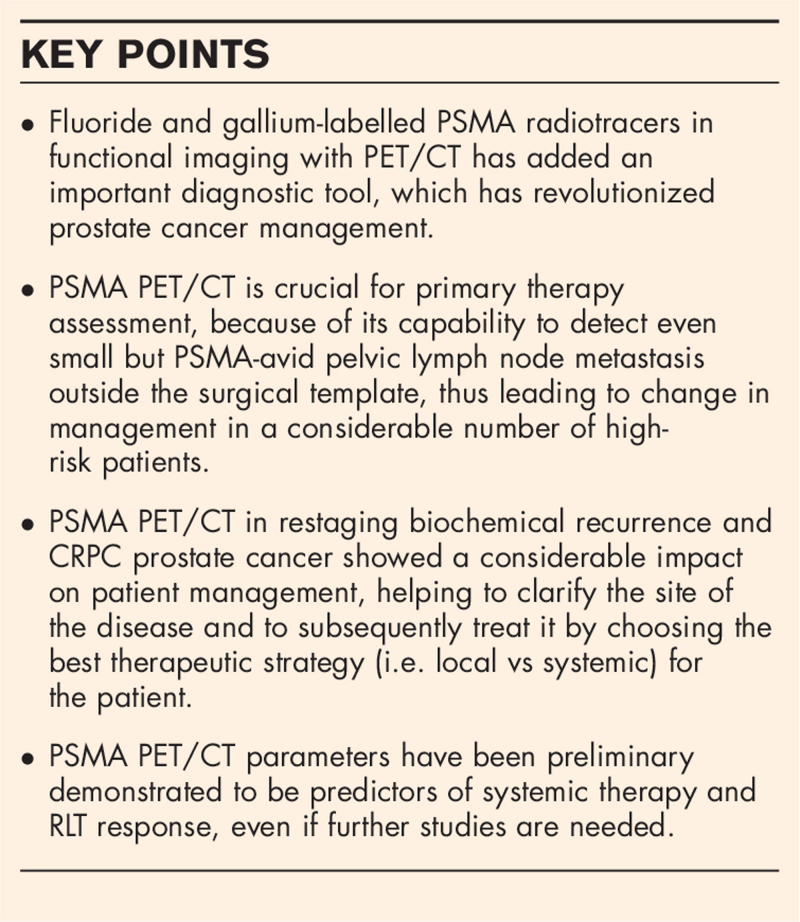
no caption available

The recent introduction of fluoride or gallium-labelled prostate-specific membrane antigen (PSMA) radiotracers in functional imaging with PET/CT has added an important diagnostic tool, which has revolutionized prostate cancer management. Firstly trialed in the setting of biochemical relapse (BCR) prostate cancer, PSMA PET/CT has since proven effective for primary staging and validated with strong prospective data [2,3]. PSMA PET/CT demonstrated superior sensitivity and specificity when compared with other conventional imaging modalities or to other PET tracers (i.e. choline, FACBC, NaF). As a result of overwhelming data evidence, PSMA PET/CT was implemented within the European Association of Urology (EAU) guidelines at staging for intermediate-to-high-risk patients and in the setting of biochemical relapse when PSA greater than 0.2 ng/ml if this can influence subsequent treatment decisions [1].

However, authors also highlight that there is still a lack of data concerning patient outcomes related to PSMA imaging-based management decisions.

Although strong data exists with regards to sensitivity and specificity in the detection of either lymph nodes and bone metastasis with various Gallium and Fluoride-labelled PSMA tracers, randomized controlled trials aimed to evaluate the real impact of the result of PSMA PET/CT the subsequent treatment choice and the related patient outcome are lacking.

The aim of this nonsystematic review is to provide an overview about the latest and most relevant data in literature about the role of PSMA ligand PET tracers in the management of prostate cancer, exploring the settings of staging, biochemical recurrence (BCR) and castrate-resistant prostate cancer (CRPC).

## THE ROLE OF PROSTATE-SPECIFIC MEMBRANE ANTIGEN IMAGING IN PRIMARY STAGING OF PROSTATE CANCER

High-risk prostate cancer is defined accordingly to EAU guidelines on the basis of several clinical factors, such as initial PSA levels, pathology grading [Gleason Score >7 or International Society Of Urological Pathology (ISUP) grade 4–5] and clinical rectal examination of at least cT2c [1]. This group represents about 25% of all prostate cancer cases and typically reflects a more aggressive disease, with shorter time to recurrence after primary treatment and even worse overall survival [4].

**FIGURE 1:**
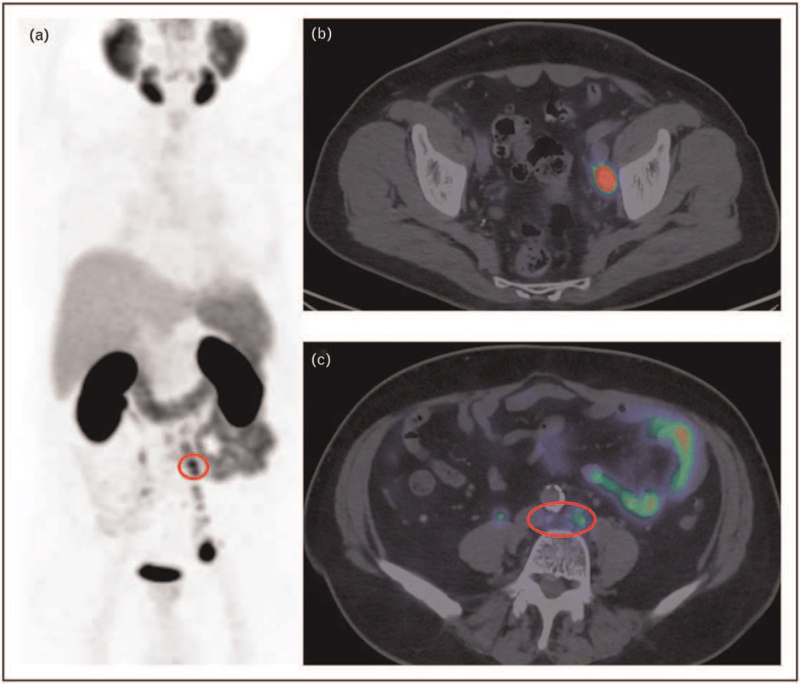
A 75-year-old man with prostate adenocarcinoma (iPSA = 26 ng/ml; GS 4+3). Primary staging ^68^Ga-PSMA PET/CT (a, MIP) showed several lymph node metastasis with increased PSMA uptake in the pelvis (b, fused images with a large left internal iliac node) and in the abdomen (c, fused image, red circle showing some small retroperitoneal nodes with PSMA uptake). Patients was excluded from surgery and subsequentley treated with ADT. ^68^Ga-PSMA PET/CT in primary staging of high-risk patients can easily detect sites of metastasis outside the surgical template, thus excluding those patients with M disease. ADT, androgen deprivation therapy; CT, computed tomography; MIP, maximum intensity projection; PSMA, prostate-specific membrane antigen.

Accurate disease primary staging with imaging is crucial for treatment decisions. The localization of pelvic lymph node metastasis in a region outside the standard pelvic dissection template (e.g. para-rectal and presacral regions) might significantly influence the surgical approach. Similarly, the detection of an extra-pelvic lymph node metastasis or a bone metastasis yields a switch to oligometastatic disease and in turn a change in treatment (from surgery to systemic therapy) (Fig. 1).

Up until recent years, conventional imaging modalities (i.e. bone scan, CT and mpMRI) were considered standard-of-care for primary staging of prostate cancer. These methods revealed poor sensitivity and specificity and were gradually overtaken by PET/CT with PSMA tracers. PSMA is a type II trans-membrane glycoprotein, which is normally expressed in some epithelia (salivary and lacrimal glands, duodenum, liver and spleen) and highly expressed on the cell surface of almost 90% of prostate cancer [5]. Moreover, PSMA expression is also correlated with tumor grade, higher PSA values and prognosis [6]. PET/CT with Ga or F-labeled PSMA tracers provided a significant increase in primary staging of prostate cancer.

A recent comparison between mpMRI and PSMA PET/CT in primary index lesion, pelvic lymph node metastasis in staging intermediate-risk and high-risk prostate cancer patients has been studied by Szigeti *et al.*[7^▪^]. A total of 81 patients were included; index lesion was detected by PSMA PET/CT and mpMRI in 87 and 98% of them, respectively. However, as regards N staging, 60% of patients with histology-proven pelvic lymph node metastasis were detected by PSMA PET/CT, whereas only 50% on mpMRI. Bone and distant lymph node metastasis were found by PSMA PET/CT in 17% of patients, of which 39% of bone metastasis appeared without any morphological correlation on CT.

The combination of PSMA PET/CT and MRI seems to be superior to MRI alone in the diagnostic performance for detecting clinical significant prostate cancer (csPC), thus potentially allowing a better selection and even a reduction in the number of prostate biopsies. In a recent prospective multicenter phase II imaging trial by Emmett *et al*. almost 300 men underwent both PSMA PET/CT and MRI prior to biopsy. Combined PSMA and MRI imaging improved NPV as compared with MRI alone [91 vs. 72%, test ratio = 1.27 (1.11–1.39), *P* < 0.001]. Sensitivity also improved, despite a slightly reduced specificity [8].

The performance of PSMA PET/CT in the detection of lymph node metastasis in primary staging of prostate cancer patients is highlighted in a recent review by Stabile *et al.*[9]. They found a moderate heterogeneity among the studies. The sensitivity, specificity, PPV and NPV of PSMA PET/CT for LNI were, respectively, 58% [95% confidence interval (CI) 50–66%], 95% (95% CI 93–97%), 79% (95% CI 72–85%) and 87% (95% CI 84–89%), with overall moderate heterogeneity between studies. Similar results were confirmed by Moreira *et al.*[10], with specificity and accuracy of 75, 96 and 91%, respectively, for lymph node involvement, and 91, 50 and 76%, respectively for metastatic bone lesions.

A recent study by Al-Ibraheem *et al.* explored the impact of ^68^Ga PSMA PET/CT on prostate cancer staging and definitive radiation therapy planning in 108 patients. Overall, PSMA PET/CT led to a change of disease stage in 36% of the patients (45% in the subgroup of primary radiation and 26% in patients intended for salvage radiation therapy). Upstaging and downstaging resulted in 22 and 14%, respectively [11^▪^].

While staging prostate cancer, the assessment of regional pelvic lymph node metastasis is crucial for surgery and subsequent clinical decisions (e.g. whether to perform PLND is pelvic lymph node dissection (PLND) or extended pelvic lymph node dissection (ePLND), subsequent salvage therapy, etc.). To better predict the risk of regional lymph node metastasis, validated nomograms (i.e. Briganti, Memorial Sloan Kettering Cancer and Winter) have been created to better select candidates for PLND. According to these nomograms, PLND should be performed in patients with lymph node metastasis risk higher than 2% (MSKCC), 5% (Briganti) and 7% (Winter) [12–14]. Despite this, more than 36% of intermediate-risk and high-risk prostate cancer patients present with nonregional pelvic lymph node metastasis (i.e. out of the PLND template) or with nonregional extra-pelvic lymph node metastasis [15]. For these patients, standard PLND might, therefore, be unnecessary as they already have disseminated disease outside the surgical template (e.g. presacral or pararectal lymph node metastasis) or even M1a disease.

In a recent study, Jiao *et al.* aimed to evaluate a threshold for current clinical PLND-validated nomograms to predict nonregional pelvic and extrapelvic lymph node metastasis in 57 high-risk prostate cancer patients studied with ^68^Ga-PSMA PET/CT. They overall observed extrareolar spread in nearly 35% of the lymph node metastasis with PSMA uptake. According to Briganti, MSKCC and Winter nomograms, 70–72% of the patients resulted in candidates to ePLND (according to EAU and NCCN guidelines). Positive lymph node metastasis on PSMA PET/CT led to a change in management in 70% of these patients. Moreover, patients with a nomogram score greater than 64, 75 and 67% according to Briganti, MSKCC and Winter, respectively were more likely to have nonregional lymph node metastasis. Authors concluded that these nomograms are able to predict nonregional lymph node metastasis. Interestingly, PSMA PET/CT may provide an additional benefit to nomograms-based clinical decision-making in more than two-third of patients for reducing unnecessary PLND [16^▪▪^].

To summarize, PSMA PET/CT is at present the best diagnostic tool for intermediate and high-risk prostate cancer patients, demonstrating excellent diagnostic performance for both N and M staging and likely superior to mpMRI in the detection of locoregional lymph node metastasis. On the basis of latest data in literature, PSMA PET/CT might be crucial for primary therapy assessment, because of its capability to detect even small but PSMA-avid pelvic lymph node metastasis outside the surgical template, thus leading to change in management in a considerable number of patients. Moreover, PSMA positivity for pelvic lymph nodes is associated with a worse prognosis in terms of BCR-free and therapy-free survival, suggesting a potential role in predicting the patient outcome and thus improving the decision about subsequent treatment, such as PLND, salvage radiotherapy and ADT.

## THE ROLE OF IMAGING IN PATIENT WITH BIOCHEMICAL RECURRENT PROSTATE CANCER

**FIGURE 2:**
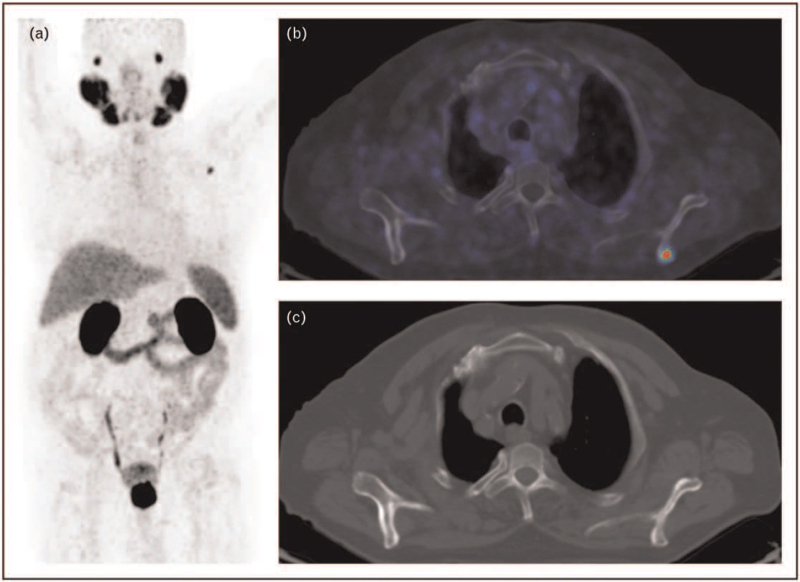
A 80-year-old man with prostate cancer (GS 4+4), previously treated with RT. At the time of the scan, PSA value was 6.4 ng/ml, with ongoing ADT. At the ^68^Ga-PSMA PET/CT scan (a, MIP) a single bone lesion in the left scapula (b, fused image) was found without any alterations on low-dose CT images (c). ^68^Ga-PSMA PET/CT can clarify the site of the disease with unprecedent accuracy and even by detecting lesions without any alteration on CT, thus helping to choose the best therapeutic strategy. ADT, androgen deprivation therapy; CT, computed tomography; MIP, maximum intensity projection; PSMA, prostate-specific membrane antigen.

Patients with PSA persistence or recurrence are at increased risk to have metastasis. When either of these occur, re-staging and subsequent appropriate management is indicated. This may be with curative intent in case of local relapse (e.g. salvage radiotherapy) or with palliative and tumor control intent in case of systemic therapy for diffuse disease spread. Restaging of these patients with PSMA imaging has become pivotal. Its superior performance compared with standard imaging [17] and to other PET tracers, is well established by a number of studies in literature, even for low PSA values [18] (Fig. 2).

In a recent review, the positive-predictive value of ^68^Ga-PSMA PET/CT according to PSA values in patients with BCR before salvage lymph node dissection ranged from 11.3 to 50% for PSA values less than 0.2 ng/ml, 20% to 72.7% with PSA 0.2–0.49 ng/ml and 25–87.5% for PSA 0.5 to less than 1.0 ng/ml [19].

Baas and colleagues retrospectively evaluated the predictive value for biochemical persistence (BCP) and early BCR of metastatic lymph node on PSMA PET/CT in 213 intermediate-risk and high-risk prostate cancer patients prior to radical treatment (RARP with ePLND). PSMA PET/CT resulted positive for lymph node metastasis in 19% of the patients, whereas pN1 was found in 23%. Sensitivity, specificity, PPV and NPV on a per-patient analysis for the detection of pN1 was 29, 84, 35 and 80%, respectively. BCP was observed in 12%, whereas early BCR in 21%. Authors concluded that a positive PSMA PET/CT prior to radical treatment was a significant prognostic tool for early relapse of the disease [odds ratio (OR) for BCP: 0.71, 95% CI 2.9-17.1; OR for early BCR: 8.1, 95% CI 2.9–22.6). Interestingly, of the 173 patients with negative PSMA PET/CT, pN1 was found in 20%, thus suggesting the importance of performing ePLND in intermediate-risk and high-risk prostate cancer patients, even with a negative PSMA PET/CT scan [20^▪▪^].

Moreover, the role of PSMA PET/CT in restaging BCR showed a considerable impact on patient management. Amiel *et al.* studied the impact of preoperative PSMA PET/CT on BCR and time to adjuvant or salvage treatment in 230 intermediate-risk and high-risk prostate cancer patients. Overall sensitivity, specificity, PPV and NPV of PSMA PET/CT for pN1 disease was around 49, 96, 82 and 82%, respectively. Biochemical recurrence-free and therapy-free survival was worse with patients with pN1 and PSMA PET/CT-positive lymph node, followed by pN1 patients with negative PSMA PET/CT (median BCR-free survival 1.7 vs. 7.5 vs. >36 months, median therapy-free survival 2.6 vs. 8.9 vs. >36 months). According to Baas and colleagues, authors concluded that patients with lymph node metastasis on staging PSMA PET/CT have an increased risk of early BCR, and are therefore, expected to undergo early adjuvant or salvage therapy [21].

Fendler *et al.* retrospectively studied with PSMA PET/CT 635 patients with recurrent prostate cancer. They overall observed a change in management for PSA values of 0.5 to less than 2.0 ng/ml in nearly 70% of them, of which approximately 50% with major changes intended as active surveillance for unknown disease site (27%), local treatment for locoregional disease (33%) or systemic therapy for metastatic disease (40%). In summary, PSMA PET/CT helps to clarify the site of the disease and to subsequently treat it by choosing the best therapeutic strategy (i.e. local vs. systemic) for the patient [22^▪▪^].

Patients who present prostate cancer relapse may benefit from different treatment modalities, whose decision needs to be well balanced by considering both the burden of the disease and the potential treatment side effects and their implications on quality of life, thus avoiding inappropriate treatments [23].

A recent study by Rogowski *et al.* aimed to explore the outcome of PSMA PET/CT-based salvage radiotherapy for lymph node recurrence in terms of BCR-free survival (BRFS) and distant metastasis-free survival (DMFS). Overall, 100 patients consecutively received sRT [with or without androgen deprivation therapy (ADT)], according to PSMA PET/CT scan results, with a median follow-up of 37 months. The following factors were predictors of better prognosis: concomitant ADT, longer ADT duration (≥12 vs. <12 months) and lymph node localization (pelvic vs. paraaortic). However, no association was observed with the number of PSMA-positive lymph nodes [24].

Generally, patients with either locally advanced (combined with radiotherapy) or metastatic disease with high risk of progression (i.e. with PSA doubling time <6 to 12 months) may benefit from ADT [1]. When patients after initial treatment with ADT experience biochemical progression, they require further re-staging PSMA PET/CT to assess the progression of the disease and eventually switch to other lines of therapy. The influence of ADT on the expression of PSMA is so far not well understood. Generally, a short period of ADT treatment seems to increase the PSMA uptake in some patients and in some prostate cancer lesions. On the counterpart, lower PSMA uptakes have been observed in long-term treatment. As the influence between ADT and PSMA expression is thus not fully understood, the decision whether to interrupt or not ADT before PSMA PET/CT is equivocal. Moreover, data in literature are limited and biased because of the lack of direct comparison of PSMA PET/CT detection rate between homogeneous population of under-ADT patients vs. treatment-naive patients [25]. At the time of this review, we found a recent study with direct comparison of the detection between two balanced groups with and without treatment. The main finding of this study is that, with comparable PSA values, the detection rate of PSMA PET/CT is significantly higher in patients on ADT than in patients off therapy. A possible explanation of these results might be more related to the advanced disease stage (i.e. to a higher tumor burden) rather than an effective influence of ADT on PSMA expression. Authors conclude that the withdrawal of ADT before PSMA PET/CT cannot be recommended [26^▪^].

However, the main limitations of PSMA PET/CT is the lack of correlation between PSMA-positive findings and histopathology, and the lack of any correlation with the patient's outcome. For this reason, prospective studies with long median follow time are required.

## THE ROLE OF IMAGING IN PATIENT WITH CASTRATE-RESISTANT PROSTATE CANCER

Patients with CRPC may benefit from a large treatment landscape aimed to delay the progression of the disease as far as possible. These therapies range from antiandrogen drugs (bicalutamide, enzalutamide, apalutamide and daroluatamide) for men with nonmetastatic CRPC (nmCRPC) to taxane-based chemotherapy (docetaxel, cabazitaxel), stereotactic radiotherapy (i.e. metastasis-directed therapy, MDT), 223Ra-dichloride, PARP inhibitors for metastatic CRPC patients (mCRPC) [27]. More recently, PSMA-targeted radioligand therapy (PSMA-RLT) have been also developed, according to the theranostic principles [28]. In these patients, it is therefore, crucial to identify the ideal moment for a change in therapy early enough to ensure the best outcome possible. This clinical aspect generates in turn the need for accurate prediction and monitoring of response. The role of PSMA PET/CT in CRPC patients is becoming predominant. Several studies demonstrated PSMA PET/CT high diagnostic performance for accurate tumor staging and patient management. Higher sensitivity has been also observed as compared with conventional imaging [29,30].

The detection rate of PSMA PET/CT in early CRPC with low PSA values has been studied by Weber *et al.* in a cohort of 55 patients with PSA less than 3 ng/ml. PSMA PET/CT resulted positive in 75% of patients and in 45% of them M1 disease was detected. For higher PSA values, PSMA PET/CT was positive in almost all the 200 patients studied by Fendler *et al.* with 55% cases with M1 disease despite conventional imaging [31^▪^].

Interestingly, Vlachostergios *et al.* evaluated the prognostic utility of PSMA uptake value in more than 200 CRPC patients who were subsequently treated with systemic therapies (chemotherapy, ADT, sipuleucel-T, 223-Ra-Dichloride). They overall found higher PSMA uptakes in 75% of the patients; moreover, they demonstrated that PSMA uptake higher than liver parenchyma is an independent prognostic factor for overall survival (OS) (hazard ratio 1.7; 95% CI 1.2–2.2; *P* = 0.003) [32].

Furthermore, the role of PSMA PET/CT parameters in predicting the response to systemic therapies in 43 mCRPC patients has been assessed by Grubmuller *et al.* Each patient received two PET-PSMA scans prior and 6 weeks after systemic therapy scans. The delta values of PET parameters were significantly associated with PSA response (dTTV *P* = 0.003, dSUVmean *P* = 0.003, dSUVmax *P* = 0.011, dSUVpeak *P* < 0001, dRECIST *P* = 0.012), whereas neither PSA values of PET parameters were associated with OS [33^▪▪^].

As regard of the impact of PSMA PET/CT in the management of CRPC patients, Fourquet *et al.* restaged 30 nmCRPC patients and observed a 100% positivity rate for high PSA values (>2 ng/ml) and 70% for PSA less than 2 ng/ml; impact of PSMA PET/CT on the management resulted in 70 and 60% for high and low PSA values, respectively [34^▪^].

PSMA PET/CT has also demonstrated a crucial impact on MDT assessment in oligometastatic CRPC patients. A systematic review by Rogowski *et al.* enlightens that PSMA PET/CT is increasingly used for staging and defining the treatment plan. However, we lack randomized data related to a better clinical outcome by using PSMA PET/CT for oligometastatic disease patients [24].

^177^Lu-PSMA RLT shows antitumor activity with an acceptable safe profile in men with mCRPC. Very recently, the TheraP trial has demonstrated a superior response in terms of PSA decrease and progression-free survival in patients receiving Lu-PSMA as compared with cabazitaxel [35]. Moreover, phase 3 VISION trial compared Lu-PSMA plus standard of care versus standard of care alone and demonstrated an improved overall survival and imaging-based progression-free survival [36]. Patients candidates to such therapy are preliminarily screened with PSMA PET/CT for PSMA expression assessment of each tumor lesion. Dual tracer PET imaging with both PSMA and FDG-PET seems to further improve patient selection for Lu-PSMA RLT, by excluding those with lower PSMA uptake or with significant PSMA-/FDG+ discordances. However, there is a lack of confirmation of FDG-PET prognostic value in a multicenter setting and a standardization of image interpretation.

Moreover, predictors of outcome after Lu-PSMA RLT are on demand. Gafita *et al.* developed nomograms to predict outcomes after Lu-PSMA RLT in more than 400 patients with mCRPC. Predictors included: time since initial diagnosis of prostate cancer, chemotherapy status, baseline hemoglobin concentration and PSMA PET/CT parameters (molecular imaging TNM (T is primary tumor, N is lymph node, M is distant metastasis) classification and tumor burden). Compared with high- risk patients, low-risk patients had significantly longer overall survival in the validation cohort [24.9 months (95% CI 16.8–27.3) vs. 7.4 months (4–10.8); *P* < 0.0001] and PSA-progression-free survival [6.6 months (6–7.1) vs. 2.5 months (1.2–3.8); *P* = 0.022) [37^▪^].

To summarize, PSMA PET/CT for CRPC patients demonstrated a considerable utility for its superior detection rate and an important impact on patient management, giving the possibility of a precise and early localization of the tumor sites and to predict the response to systemic or radiotherapy-targeted therapies. Moreover, with regards to theranostics, PSMA PET/CT is pivotal for selecting the patients eligible for Lu-PSMA RLT, also demonstrating (although only preliminary) a predicting value in the assessment of therapy response.

## CONCLUSION

PSMA PET/CT imaging has rapidly become a fundamental tool for staging and restaging prostate cancer. Its unprecedent sensitivity allows to better customize the treatment for each patient at any stage of the disease. The potential to guide therapy and to early identify relapses, as well of the impact on patients’ outcome is being explored, although further studies are needed aimed to assess its impact on overall survival. For advanced metastatic prostate cancer, PSMA imaging is critical for selecting patients suitable for RLT and promising data suggest a possible role as predictor of response.

## Acknowledgements

*None.*

### Financial support and sponsorship

*None.*

### Conflicts of interest

*There are no conflicts of interest.*

## REFERENCES AND RECOMMENDED READING

Papers of particular interest, published within the annual period of review, have been highlighted as:

▪ of special interest
▪▪ of outstanding interest
